# Development of High Resolution Melting Analysis as a Diagnostic Tool for Molecular Detection of *Toxoplasma* Infection in Pregnant Women and HIV Positive Cases

**DOI:** 10.18502/ijph.v49i10.4704

**Published:** 2020-10

**Authors:** Ahmad HOSSEINI-SAFA, Saeedeh SHOJAEE, Seyed Alireza SALAMI, Mehdi MOHEBALI, Sedigheh HANTOUSHZADEH, Parisa MOUSAVI, Seyed Ali DEHGHAN MANSHADI, Hossein KESHAVARZ VALIAN

**Affiliations:** 1. Department of Medical Parasitology and Mycology, School of Public Health, Tehran University of Medical Sciences, Tehran, Iran; 2. Department Biotechnology, School of Agricultural Sciences and Engineering, University of Tehran, Tehran, Iran; 3. Center for Research of Endemic Parasites of Iran (CREPI), Tehran University of Medical Sciences, Tehran, Iran; 4. Maternal, Fetal and Neonatal Research Center, Vali-Asr Hospital, Imam Khomeini Hospital Complex, Tehran University of Medical Sciences, Tehran, Iran; 5. Skin Diseases and Leishmaniasis Research Center, Isfahan University of Medical Sciences, Isfahan, Iran; 6. Department of Infectious Diseases and Tropical Medicine and Iranian Research Center for HIV/AIDS, Tehran University of Medical Sciences, Tehran, Iran

**Keywords:** High-resolution melting (HRM), Toxoplasmosis, Pregnant women, HIV^+^ patients

## Abstract

**Background:**

*Toxoplasma gondii* is an obligate intracellular protozoan with worldwide distribution. Diagnosis of toxoplasmosis is a very critical issue, especially in pregnant women and immunocompromised patients. The aim of this study was rapid detection of *T. gondii* DNA in peripheral blood samples (PBS) employing HRM technique and using RE gene.

**Methods:**

Totally, 242 samples from pregnant women and human immunodeficiency virus (HIV) patients were collected from different hospitals and medical centers of Tehran during Oct 2017 to Dec 2018. High resolution melting analysis (HRM) using partial sequences of repetitive element (RE) gene was done and compared with ELISA test.

**Results:**

Overall, 51 were positive for acute toxoplasmosis that among them, 12 and 20 reported as positive in pregnant women and HIV^+^ patients, respectively using HRM technique. Among 70 patients in chronic phase of disease, 10 and 3 samples were reported as positive for pregnant women and HIV^+^ patients respectively. From 121 negative control, 3 (4.62%) samples associated with HIV^+^ patients, showed positive real-time PCR and HRM analysis results.

**Conclusion:**

For the first time, HRM technique via employing RE gene was used for detection of *T. gondii* infection in PBS. This method is suitable, helpful and in parallel with serological methods for early diagnosis of acute as well as active form of toxoplasmosis in pregnant women and HIV^+^ patients. The use of techniques based on melt curve and through employing next-generation dyes for diagnosis of *T. gondii* would be accessible for patients in developing countries.

## Introduction

*Toxoplasma gondii* is an obligate intracellular protozoan parasite with a global prevalence (1). Almost all warm-blooded creatures, including humans can be infected with this parasite (2–4). Around 30% of the human population around the world will experience at least one chronic infection with this parasite throughout their lives (5, 6). In immunocompetent individuals, clinical symptoms of toxoplasmosis are mild and self-limiting including malaise and lymphadenopathy as well as fever. However, these symptoms are severe and associated with more complications including retinochoroiditis, encephalitis, fetal abortion, splenomegaly, and pneumonitis in those with immunocompromised conditions including AIDS, which may turn into a disseminated form (7–12). In recent studies, *T. gondii* has also been implicated as a risk factor for mental disorders, such as schizophrenia and Alzheimer’s disease (13–15).

Diagnosis of toxoplasmosis is a very critical issue. Immunological methods based on identifying IgG and IgM against *T. gondii* and avidity tests are among the major means used for differentiating between acute and chronic phase of the disease (16, 17). Concerning the intrinsic limitations of methods based on serological assay such as limitation of antibody detection in immunocompromised patients, polymerase chain reaction (PCR) assay can play a significant role as a supplement method in diagnosing of toxoplasmosis (5, 18, 19).

So far, various molecular diagnostic methods have been used for tracking the parasite DNA in biological samples of human body. They include conventional methods as well as methods based on taqman real-time PCR (20–22). The conserved genes of *T. gondii* that are usually used in molecular detections (PCR) include B1 gene with 35 copies as well as repetitive region (RE) with a length of 529bp and 300 copies in the genome of the parasite (16, 23). Among some of the advantages of HRM technique can be noted as rapid, low-cost, and sensitive detecting method to identification of DNA using a single step PCR (24). No study has been done using high-resolution melting analysis (HRM) technique for diagnosing of toxoplasmosis in the peripheral blood samples (PBS) of patients with HIV and pregnant women, while all the studies employing this technique have dealt with genotyping of the parasite.

We aimed to identify the DNA of the parasite in the PBS samples of pregnant women and immunocompromised patients with toxoplasmosis through applying HRM technique and using RE gene comparing to IgM and IgG ELISA test.

## Materials and Methods

### Patients and clinical samples

Blood samples were collected from different hospitals and medical centers of Tehran during Oct 2017 to Dec 2018. After routine serological screening in hospitals and medical centers, suspicious samples from two groups including pregnant women (group I) with 19 samples related to anti-*T. gondii* IgM and IgG antibodies and 37 samples with anti-*T. gondii* IgG antibody were collected. Samples were also collected from HIV positive patients (group II) including 32 samples with positive anti-*T. gondii* IgM and IgG antibodies and 33 samples with anti-*T. gondii* IgG antibody. From each patient 2ml PBS for serological tests and 2ml of whole blood samples for DNA extraction in the presence of ethylene-diaminetetraacetic acid (EDTA) were collected. All the patients in group II had a confirmed previous history of HIV infection with CD4 cell counts < 200 cells/mm^3^. Moreover, 56 and 65 seronegative whole blood samples for anti-*T. gondii* antibodies were collected from pregnant women and HIV patients respectively, as negative controls.

### ELISA

A part of whole blood samples was centrifuged and afterward serum samples collected and stored for a maximum time of 24 h at 4 °C until serological analysis. Anti-*T.gondii* IgG and IgM antibodies were evaluated by *Toxoplasma* IgM and IgG kit (Pishtaz Teb Zaman Diagnostics, Iran). The ELISA test has been done according to the manufacturer’s instructions (25).

### DNA extraction from patient samples

Total genomic DNA was extracted from whole blood of group I, group II, and negative control samples using high pure PCR template preparation kit (Roche, Germany) according to the manufacturer’s instructions with some modifications. The concentration of the extracted DNA was measured by NanoDrop (Thermo Scientific, Rockford, IL, USA). Finally, the samples were kept at −20 °C for real-time PCR and HRM analysis.

### Real-time PCR and HRM analysis

The partial RE sequence gene was amplified using specific primers, forward A.H.S-TOXO-F 5’- CTGCGTCTGTCGGGATGA-3’ and reverse A.H.S-TOXO-R 5’- GCGTCGTCTCGTCTGGAT-3’ design was carried out using the sequences available on GenBank (accession number: AF146527.1). Then, primers were obtained using the Beacon Designer8.12, PREMIER BIO-SOFT software and rechecked with PRIMER BLAST software (http://www.ncbi.nlm.nih.gov/tools/primer-blast). Consensus sequence was obtained from the multiple sequence alignments, with a predicted amplicon size of 321 bp for the sequence. Real-time PCR and HRM analysis was performed in 20 mL final reaction volume containing 4 mL master mix HOT FIREPol EvaGreen HRM Mix (ROX)(Solis BioDyne Co, Estonia), 10.2 mL molecular grade water (DNase and RNase free water, SinaClon BioScience Co, Iran), 0.4 mL of each HPLC purified primers, and 5 mL of template DNA. The real-time PCR amplification was performed as follows: for initial denaturation, the reaction mixture was heated at 95 °C for 5 min, followed by 40 cycles of amplification performed at 95 °C for 25 sec for denaturation step, 58 °C for 30 sec, for annealing section, 72 °C for 30 sec for extension portion, and final holding extension stage at 72 °C for 5 min after 40 cycles was performed. Then, HRM was performed at 95 °C for 15 sec while raising temperature from 75 to 95 °C. During this process, the amplicons obtained from PCR were denatured before the development of melting curves in the inflexion point, where changes in fluorescence concerning changes in temperature (dF/dT) were recorded with a ramp of 0.3 °C/sec (26). Fluorescence dye signaling was measured after each cycle. The kit contained the next generation-stranded DNA-binding fluorescent dye, EvaGreen, and an optimized HRM PCR master mix buffer, consisting of HotStarTaq plus DNA polymerase, Q-Solution, and dNTPs.

In the current study, the samples used as negative control (NC) had negative results for IgM and IgG antibodies and with non-template control samples (NTC) was used in each real-time PCR and HRM analysis running. Moreover, a positive standard control of tachyzoites of *T. gondii,* RH strain was used. Tachyzoites were obtained from Toxoplasmosis Reference Laboratory of the Department of Medical Parasitology and Mycology, School of Public Health, Tehran University of Medical Sciences. Real-time PCR was carried out in a Mini Opticon real-time PCR detection instrument (Applied Biosystems Step One Plus Inc., CA, USA). The real-time amplification results and T_m_ analysis were obtained using the Step One Plus^TM^ software Ver. 2.3 (Life technologies^@^). To confirm the results and for repeatability, each run was repeated for three times. Another reason was estimating the T_m_ variations within a PCR amplification (intra-assay) and between PCR amplifications (inter-assay). The coefficient of variation (CV) was calculated by dividing the standard deviation (SD) by the arithmetic mean of the measured values of Tm (CV = [SD]/mean value). Further, to check the temperature uniformity in the cycler block, during the same amplification cycle, some samples were re-amplified at different positions of the cycler block. The intra-assay CVs represent the mean CVs of the results obtained from the replications of *T. gondii.*

### Ethical statement

The research project was approved and reviewed by the Ethics Committee and Vice Chancellor for Research, Tehran University of Medical Sciences, Tehran with approval code: (No: IR.TUMS.SPH.REC.1397.008).

## Results

### ELISA

Out of 242 samples from pregnant women and HIV^+^ individuals, 51 had anti-*T. gondii* IgM antibody, accounting for 21% of all samples of patients. Out of them, 19 samples were associated with pregnant women and 32 were observed in HIV^+^ patients. Furthermore, out of 70 samples of patients with anti-*T. gondii* IgG^+^/IgM^−^, 37 and 33 samples were related to pregnant women and HIV^+^ patients, respectively. In this study equivalent to the entire sample collected with positive IgM and IgG antibody titer, negative controls were collected in each group, that out of 121 individuals with anti-*T. gondii* IgM^−^/IgG^−^, 56 and 65 samples were related to pregnant women and HIV^+^ patients, respectively (Table 1).

**Table 1:** The results of ELISA test along with real-time PCR and HRM assay for detecting acute and chronic form of *T. gondii* in pregnant women and HIV^+^ patients

***Patient Group***	***ELISA results***		***Results of HRM analysis***		

	***No. of patient (%)***		***Positive result***		***Negative result***
			***No. of patient (%)***	***Mean Ct***	***No. of patient (%)***
Group I	IgM+ / IgG+	19 (100)	12 (63.15)	23	7 (36.85)
Pregnant women	IgM− / IgG+	37 (100)	10 (27)	28	27 (73)
	IgM− / IgG−	56 (100)	0 (0)	---	56 (100)
Group II	IgM+ / IgG+	32 (100)	20 (62.5)	23	12 (37.5)
Immunocompromised patients	IgM− / IgG+	33 (100)	3 (9%)	28	30 (91)
	IgM− / IgG−	65 (100)	3 (4.62)	28	62 (95.38)

### Real-time PCR amplification and HRM analysis

**Fig. 1:**
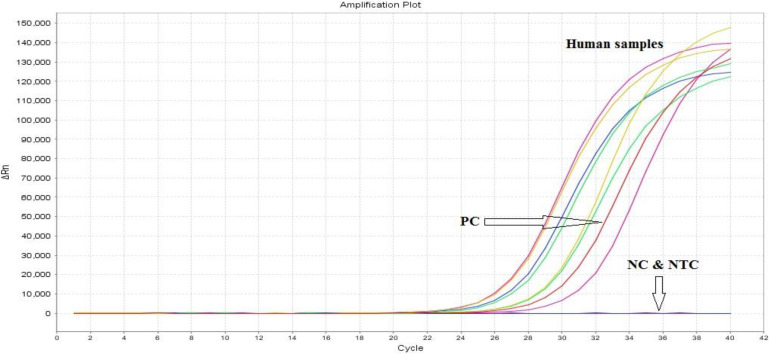
Amplification plot of *T. gondii* in human whole blood samples with positive control (PC), negative control (NC) and non-template control (NTC) using RE gene sequence

**Fig. 2:**
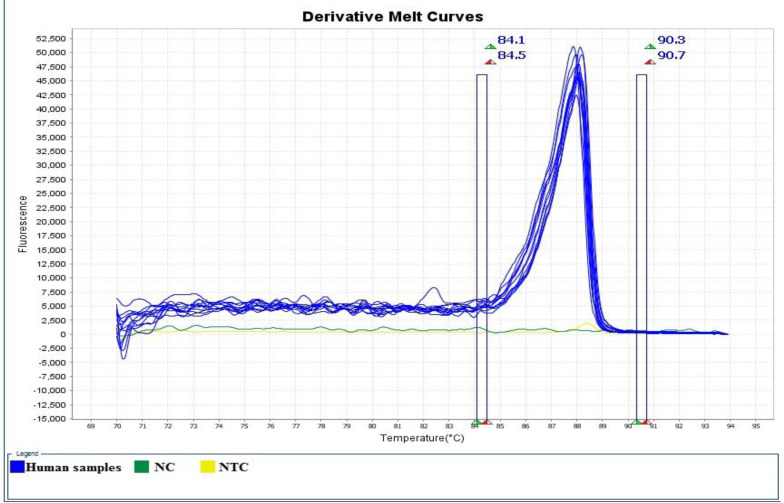
Derivative melt curve for RE amplicon showing melt curves analyses of *T. gondii* in human blood samples with positive control (PC), negative control (NC) and non-template control (NTC) samples

**Fig. 3:**
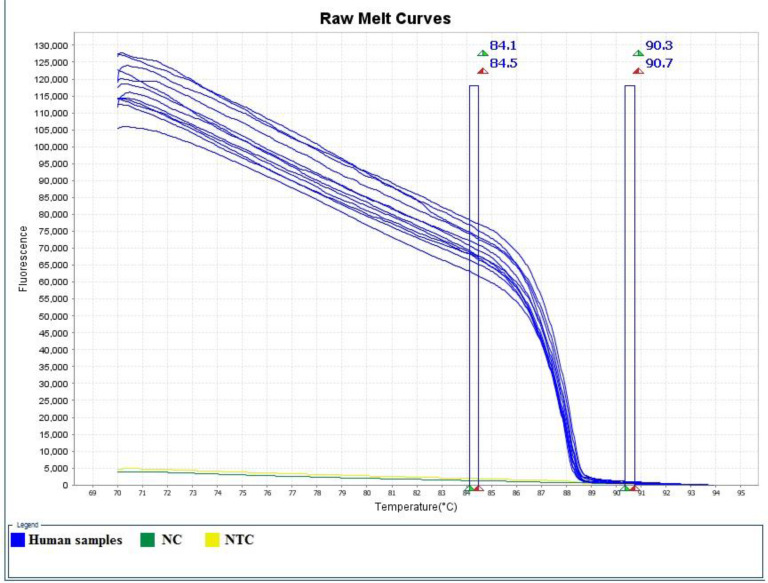
HRM plots for RE amplicon showing melt curves analyses of *T. gondii* in human blood samples with positive control (PC), negative control (NC) and non-template control (NTC) samples

The results of real-time PCR and HRM analysis showed that out of 51 patients with acute phase of the disease, 63.15% and 62.5% were reported as positive for pregnant women and HIV^+^ patients, respectively, whose mean Ct has been 23. Furthermore, out of 70 patients in chronic phase of disease, 27% and 9% were reported as positive for pregnant women and HIV^+^ patients respectively according to real-time PCR and HRM analysis, whose mean Ct has been 28. In this study out of 65 negative control individuals associated with HIV^+^ patients, 3 (4.62%) showed positive real-time PCR and HRM analysis results (Table 1). The amplification plot, derivative melt curve and HRM melt curve analysis of the *T. gondii* are displayed in Figs. 1–3. The HRM curve results representing the mean CV, SD, and T_m_ calculated based on inter- and intra-assay are reported in Table 2.

**Table 2:** Mean SD, CV, and T_m_ calculated based on intra- and inter-assay of RE gene sequence of *T. gondii*

***Gene***	***SD***	Intra-assay CV^*^ (%)	***Inter-assay CV (%)***	***Mean Tm (°C)***
RE				
*T. gondii*	0.12	0.07	0.10	88.1°C

## Discussion

The final and definitive diagnosis of toxoplasmosis and precise differentiation of acute and chronic infections is critical nowadays. Although serological techniques can identify IgM antibody, high value of IgM antibody titer does not always signify the existence of acute infection, since IgM antibody titer may remain in the patient’s serum years after active infection. In such conditions, avidity test may be able to differentiate between chronic and acute phase by drawing a distinction between IgG low and high affinity. This is especially crucial in immunocompromised patients as well as in pregnant women for whom rapid and definite diagnosis of the disease can change the therapeutic strategies. In such a scenario, molecular tests can play a significant role in diagnosing of acute infections by detecting and tracking presence of parasite in the bloodstream or other clinical samples. Direct detection of DNA of *T. gondii* in the blood and other clinical samples can confirm the presence of the parasite in the patient’s body resulted in development of acute primary infection, reactivation, or chronic infection especially in immunocompromised patients (18, 20).

So far, various samples of body fluids have been used for molecular detection of toxoplasmosis (7, 20, 27). Furthermore, various molecular techniques including conventional PCR and real-time PCR have also been employed for diagnosis of toxoplasmosis. In different studies, typically B1 and RE major genes have been used for clinical diagnosis given the more copies in the parasite genome. Concerning the larger number of copies in the genome of the parasite, RE sequence with the length of 529bp in real-time PCR technique has greater efficiency in comparison to B1 gene in diagnosis of toxoplasmosis (27). Due to existence of more copies in the RE gene, this gene was chosen in this study. Among the advantages of methods based on real-time PCR over conventional methods one can mention the sensitivity and specificity of these methods, being closed tube and time-efficient (26, 28, 29). Eventually, we can conclude that real-time PCR methods can detect fewer copies of the parasite genome in comparison with conventional methods. Concerning the nature of the use of next-generation dyes in HRM technique such as Eva Green, LC Green, and SYTO 9 Green which have a high saturating level and lower inhibition value in replication of PCR in comparison to SYBR Green dye, typically used in real-time PCR melt curve, this issue has a direct effect on greater diagnostic sensitivity and the saturating level of DsDNA in techniques in which these dyes are used. Therefore, usage of next-generation dyes may be able to better represent fewer replications in the parasite genome in the PCR test process (30–32).

So far, various studies have been performed for genotyping of *T. gondii* using HRM technique, including the study with using B1 gene (33, 34). Moreover, different studies have been performed to detect non-human samples using HRM technique, including the study using HRM technique that they dealt with detecting of *T. gondii* and *Cyclospora cayetanensis* in *Mytilus galloprovincialis* (35). Further, in another study, dealt with identifying *Cryptosporidium* and *T. gondii* as well as *Giardia lamblia* in wastewater and oyster samples in Italy and Turkey through multiplex PCR and HRM assay (36). This technique using human samples (tears and eye rinse liquid) for detection of parasite in ocular toxoplasmosis was used in one study (37). Eventually, in our study for the first time, HRM technique has been used to identify and detect the parasite DNA in the blood sample of patients. In this study, among the pregnant women who had positive IgM and IgG antibody, 63.15% and 23% had also positive HRM test results. Furthermore, among HIV^+^ patients with positive IgM and IgG antibodies the HRM results were reported as 62.5 and 9%, respectively. These results are similar to the studies used taqman real-time PCR for detecting the DNA of *T. gondii* in blood samples. One of these studies used B1 gene to detect the DNA of the parasite in PBS of patients. They reported that out of the individuals with IgM^+^ antibody, only 48.5% had positive result. Furthermore, out of the patients with IgG^+^, only 3.6% were reported as positive and they also reported three positive DNA amplification among 38 negative control samples using taqman real-time PCR (20).

In our study from 65 negative control individuals associated with HIV^+^ patients, 3 (4.62%) showed positive real-time PCR and HRM analysis results. This finding likely reflects the presence of circulating *Toxoplasma* in the bloodstream without the presence of antibodies. Similarly, in another study using taqman real-time PCR and B1 and RE genes in pregnant women, 68% and 25% as IgM^+^ and IgG^+^ were detected respectively. Moreover, in immunocompromised patients, the positive values were 48.5% and 7.4%, respectively for taqman real-time PCR, which are similar to the results obtained from this study (27).

HRM technique in terms of diagnostic power, it is equivalent to taqman probe methods. Use of taqman real-time PCR such as MGB taqman probe methods probably offer greater sensitivity and specificity compared to melt curve methods. Therefore, use of melt curve methods, concerning their close and acceptable specificity and sensitivity compared to taqman methods can significantly help in diagnosis of toxoplasmosis. Future studies are performed with a larger sample size of patients through HRM and using RE gene for detecting congenital toxoplasmosis.

## Conclusion

HRM technique via employing RE gene is suitable, helpful, and in parallel with serological methods for early diagnosis of acute as well as active form of toxoplasmosis in pregnant women and HIV^+^ patients. In the not-too-distant future use of techniques based on melt curve and through employing next-generation dyes for accurate diagnosis of *T. gondii* would be accessible.
